# Toward a Standard Measure of Abortion Service Quality-A Stakeholder First Approach

**DOI:** 10.3389/fgwh.2022.903914

**Published:** 2022-07-04

**Authors:** Nirali M. Chakraborty, Erin Pearson, Caitlin Gerdts, Sarah E. Baum, Bill Powell, Dominic Montagu

**Affiliations:** ^1^Metrics for Management, Baltimore, MD, United States; ^2^Ipas, Chapel Hill, NC, United States; ^3^Ibis Reproductive Health, Oakland, CA, United States

**Keywords:** abortion, quality of care, measurement, user-centered design, stakeholder acceptance

## Abstract

Measurement of the quality of abortion services is essential to service improvement. Currently, its measurement is not standardized, and some of the tools which exist are very long, and may deter use. To address this issue, this study describes a process used to create a new, more concise measure of abortion care quality, which was done with the end users in mind. Using a collaborative approach and engaging numerous stakeholders, we developed an approach to defining and selecting a set of indicators, to be tested against abortion outcomes of interest. Indicators were solicited from 12 abortion service provision entities, cataloged, and grouped within a theoretical framework. A resource group of over 40 participants was engaged through surveys, webinars, and one in-person meeting to provide input in prioritizing the indicators. We began with a list of over 1,000 measures, and engaged stakeholders to reduce the list to 72 indicators for testing. These indicators were supplemented with an additional 39 indicators drawn from qualitative research with clients, in order to ensure the client perspective is well represented. The selected indicators can be applied in pharmacies, facilities, or with hotlines, and for clients of surgical or medical abortion services in all countries. To ensure that the final suggested measures are most impactful for service providers, indicators will be tested against outcomes from 2,000 abortion clients in three countries. Those indicators which are well correlated with outcomes will be prioritized.

## Background

High quality, client centered health services are a human right, and access to the full spectrum of reproductive health options is necessary for all people to achieve that right. A critical component of reproductive health care is safe, high quality abortion services. In settings where abortion is legal, its safety has been well established (1). Yet, what constitutes a quality healthcare service, both broadly speaking (2–4), and for abortion services in particular (5–8), does not have a standard definition. Of more immediate pragmatic interest for clients, regulators, and practitioners, abortion quality lacks a common measure to evaluate or improve quality, regardless of the definition used.

When evaluating the quality of a particular health service, such as abortion care, it is important to be able to compare across geographic areas, providers, or modes of care. Comparison allows providers to evaluate their performance, and clients or policy makers to understand the range of options for services that are possible.

Significant strides have been made to measure the quality of abortion services by researchers as well as service delivery organizations in a variety of settings (9–11). However, comprehensive, existing measures are varied and not standardized (8, 12). Focusing on published papers and reports of abortion quality alone, Dennis and colleagues found 56% of indicators cited in only a single source, while Filippi and colleagues' broader net of abortion measures found only 22% of indicators in their review were cited across multiple sources.

Both the World Health Organization (WHO) quality of care framework and WHO's vision for maternal and child health emphasize that the experience of care is an essential component of quality (4, 13, 14). Yet, measurement of the client's experience in abortion care is limited, and often focused solely on client satisfaction (6, 8, 12, 15). Defining and including client experience measures is important for a comprehensive understanding of quality care (16–18).

Development of a set of standard measures to assess abortion service quality, irrespective of the service delivery channel (facility, pharmacy, hotline) or procedure type (medical, surgical) can ensure that a wide array of providers can efficiently and comparably collect data on and strengthen their services. To date, there are no studies which compare the quality of abortion services across different channels, or procedure types. Yet, pregnant people should receive the same level of quality care irrespective of the location and type of their legal abortion. Understanding how that can be measured is a valuable addition to the monitoring and evaluation already being conducted by many large non-profit service providers. Furthermore, it will aid public sector and for-profit private sector care providers, who lack the resources for extensive clinical evaluations.

Despite this assertion, the authors' goal in creating a new measurement tool is utilization. To ensure a wide array of possible needs and voices are included, members of the Abortion Service Quality Initiative (ASQ Initiative) have led a collaborative effort with key partners, donors, and Ministries of Health to achieve this goal. This effort is modeled after other consensus driven and stakeholder inclusive methods, including the over 80 articles which have used a Delphi process or modified Delphi process to select quality indicators (19–22). Specifically for the selection of quality indicators, Campbell and colleagues note that different stakeholder groups have different foci of quality (21). They and others note that a process that combines evidence with consensus can facilitate the development, visibility and validity of decisions in areas of uncertainty or incomplete evidence (20, 21).

This paper describes the selection of indicators to be tested for a simplified and standard suite of measures for abortion service quality.

## Methods

The process the ASQ Initiative undertook for selecting indicators to pilot test for a simple measure of quality began in 2017 by establishing consensus on a need for a more simple measure. It has advanced through progressive stages of indicator identification, consolidation, reduction, and testing following the Metrics for Management metric development process (7). Throughout, the process has engaged international stakeholders in the guidance and review of decisions for indicator inclusion, identification of outcomes for indicator validation, and engagement in formulating the priorities and processes for reduction. Collective decision-making and transparency were prioritized from the start.

### Consensus on Need

As described above, Dennis et al.'s review of measures for abortion quality concluded that there was little or no agreement on definitions or indicators in the field (12). This article was published in 2017, and in April of that year an expert group gathered with the aim of defining the challenges in quality measurement specifically for service implementation, and building consensus for a possible solution. The three-day meeting was attended by 44 participants from 16 countries, who represented large and small service delivery agencies, international institutions, funders, ministries of health and academic researchers. Meeting participants discussed measurement of quality for abortion services, across a range of delivery contexts, and procedure types. They agreed that greater standardization would be welcome, useful, and should prioritize applicability in low resource settings. Further, the indicators should be actionable, valid, accurate, timely, simple and easy to collect. These priorities were considered throughout the process. In presenting measurement tools from their own organizations, participants noted the lack of indicators to measure patient experience, and the insufficient measurement tools assessing pain and discomfort. Following this meeting, the participants, as both individuals and representatives of organizations, became the founding members of a stakeholder representation group, organized under the name the “ASQ Resource Group” (ASQ RG). ASQ RG members were envisioned to participate in the indicator development process and dissemination, by providing insight at key decision making points. Three non-profit organizations, Metrics for Management, Ibis Reproductive Health, and Ipas, joined together to lead the Abortion Service Quality Initiative (ASQ), and are the research team referred to below.

### Review of Indicators and Evidence

As described in previous publications, the ASQ Initiative began with a review of existing frameworks and indicators, and then adopted key components from two existing frameworks to structure measures and indicators for abortion quality (7). First, the six dimensions of quality (Effective, Efficient, Acceptable, Accessible, Equitable, Safe) set out by the Institute of Medicine, and adapted to maternal and reproductive health by the WHO (3, 13); and second, the categorization proposed by Dennis et al. (12). Application of the resulting framework first identified an indicator as measuring structural or process quality, output or outcomes; and then assigned each indicator to a subtheme derived from the IOM/WHO model.

Following the consensus building meeting, 10 organizations representing abortion service delivery in both low- and middle-income countries (LMIC), along with two LMIC Ministries of Health, shared the quality assessment tools and indicators they use to monitor abortion quality. The development and testing of process indicators of quality is, in itself, a multi-stage endeavor, and having a starting point of tested tools allowed the team to leapfrog a few steps (23). With these tools as a starting point, the research team undertook a series of steps to define a unique list of indicators. The research team cataloged all indicators from the 12 organizations, creating a database which contained information on the source, numerator, denominator, collection frequency, data collection tool used, if the indicator is derived from other data, and whether and why it is recommended for exclusion. Each indicator was categorized according to the WHO dimension of quality (Effective, Efficient, Acceptable, Accessible, Equitable, Safe). Next, we attempted to categorize indicators to match Dennis et al.'s 75 unique indicators of quality abortion care (12). In most cases, one of the indicators from the ASQ database was linked to multiple indicators from the Dennis et al. list. Each organization's materials were coded independently by two researchers, and if consensus was not achieved, it was brought to the larger ASQ research team for adjudication. Reasons for exclusion were that the item was not about quality (i.e., clinic revenue) or that it was not measured or amenable for change at the facility level (i.e., laws permitting abortion). Some difficult to classify indicators which were related to multiple indicators within the Dennis et al. paper were included or excluded on a case by case basis.

### Assignment to Domains and Alignment of Existing Indicators

**Figure 1:**
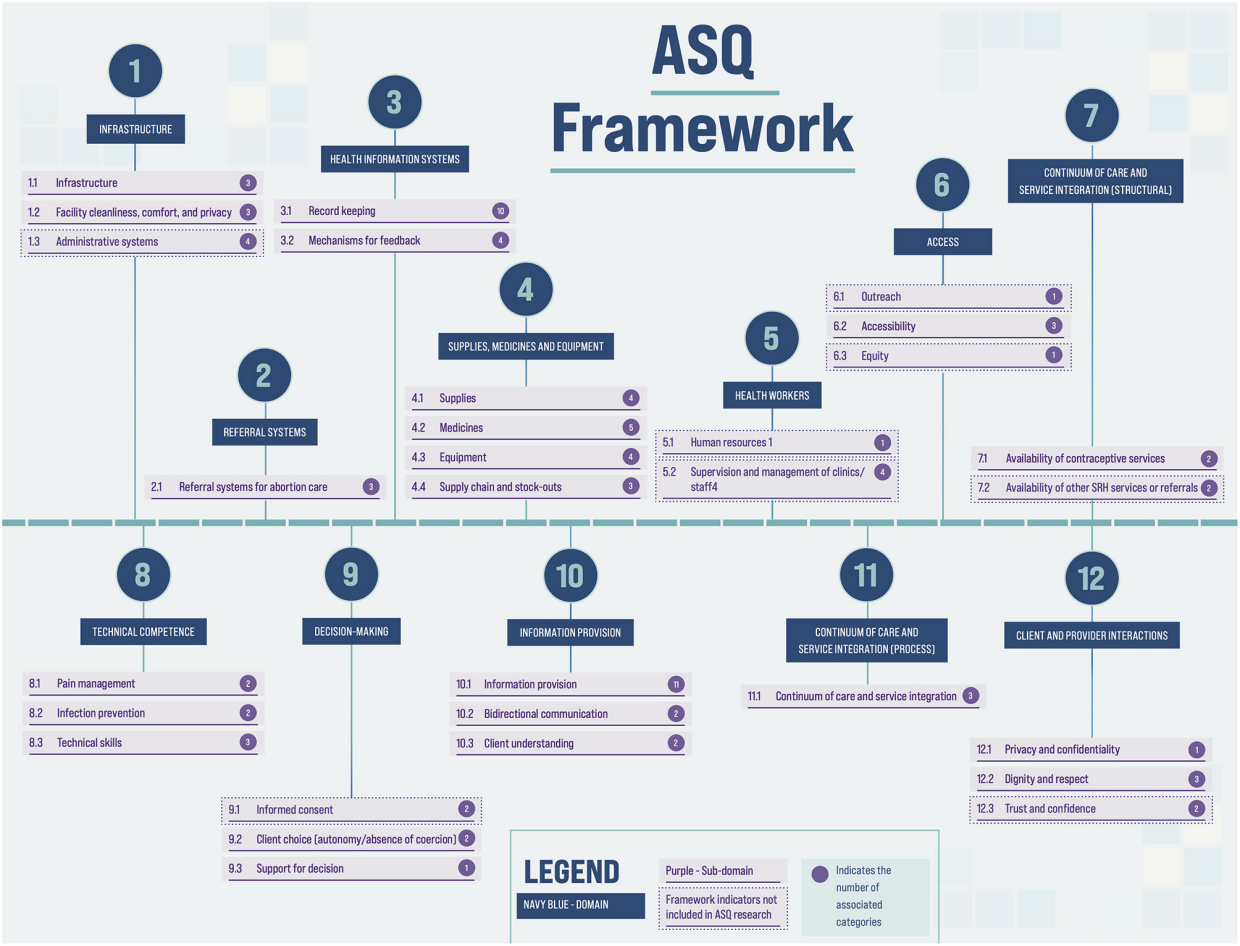
ASQ indicator framework.

Further categorization was required before a unique list could be defined. To accomplish this a more granular framework for categorization was inductively created, drawing from the sub-categories outlined in Akachi and Kruk (24), as well as from a review of the indicators. Via consensus among six researchers representing the three ASQ partner organizations, indicators were assigned to a domain and sub-domain (see Figure 1). Next, indicators were assigned to a “category” – a third level of the framework. Within each category, duplicate indicators with identical or near-identical wording were identified, and one indicator was kept as best representing the duplicates. For this process, identified duplicates were reviewed by another team member before any were dropped.

Finally, as some organizations collected individual data elements, whereas others collected more broadly worded indicators, a final set of unique indicators was created by combining indicators or elements with similar intent together and rewording if needed (see Box 1 for examples).

Box 1 **Consolidation of indicators with similar intent.**

*Example 1*: Two indicators assess if “Client is greeted with a smile” – one via direct observation, and another via client exit interview. Indicators are combined, and both possible methods of inquiry are noted.

*Example 2*: Three unique items assess if a client is given a) dressing gown, b) socks and c) cloth drape. These granular and context specific measures and are grouped and reworded as “Clothing for client privacy and comfort”.

### Reduction

The resulting ASQ conceptual framework contained almost 100 categories and over 200 unique indicators. In order to further reduce the list, we fielded two consecutive online surveys to seek input from members of the ASQ RG. In the first survey, in December 2018, the ASQ RG members were given a list of the categories in the framework, placed within the framework hierarchy of domains and sub-domains. They were asked to indicate which categories they believed to be “most critical to measure well, in order to provide high quality abortion services.” Respondents were also asked to provide information about the rationale used in their selection process. Categories selected by at least 25% of respondents to the survey were retained at this stage.

The second survey took place in February 2019, and focused on refining indicators within the retained categories. Several of the retained categories contained 10 or more unique indicators; additionally, some indicators contained lengthy checklists of supplies required, or steps in a process; attributes which would make them difficult to collect. The ASQ RG were asked to prioritize a limited number of indicators within these categories, and/or a limited number of items within an indicator checklist. Examples of these reduction requests are shown in Box 2. Indicators which were selected by 50% of respondents were retained after this round of external input. For both surveys, RG members were made aware of the survey via a webinar or meeting in advance of its launch, and received at least three electronic invitations to participate.

Box 2 **Selection and reduction of indicator categories and checklists.**

*Example 1*: For each indicator related to “Integration of SRH services” in the list below, is this necessary to measure in a standardized way, in order to improve abortion service quality? The list included 6 indicators.

*Example 2*: For each item related to “Supplies for exams and procedures” in the list below, is it necessary to measure the presence of this item in the consultation room only, in the operating theater only, in both, or not necessary in either location? This question is administered using a standardized checklist whose length could be reduced. The list included 25 items.

### Addressing Gaps in Existing Quality Measurement

Given the limited measures of client experience in existing tools and consensus at the expert meeting in 2017 that client experience is a critical aspect of quality, we conducted formative research with abortion clients at abortion facilities, hotlines, and unlicensed drug sellers in four countries. Through seven focus groups and nearly 100 in-depth interviews of people seeking abortion in Argentina, Bangladesh, Ethiopia and Nigeria, we sought to understand those features of the abortion service experience which mattered most to them (25). To develop indicators, we created a codebook to code *in vivo* and a priori quality domains and applied the codebook to the interviews. We drafted code summaries for each quality domain and then drafted indicators based on the patterns that emerged in each code summary. These newly drafted indicators were reviewed alongside the list of indicators derived from the 12 service delivery organizations, and duplicates in the existing ASQ database were discarded. Where those concepts were already measured by indicators in the existing ASQ database, but from a different perspective (eg: service provider rather than client) the research team included both measurement methods.

### Identifying Outcomes for Indicator Validation

Finally, a subset of the ASQ RG was engaged to define the outcome(s) against which indicators of quality would be assessed. The group worked virtually over a period of weeks to brainstorm, review and define outcomes for assessment. All indicators and outcomes were operationalized for pilot testing, through the design of appropriate study tools, and defining which clients and sites were eligible respondents.

## Results

Starting with data collection tools and resources from 12 public and private abortion service delivery organizations, we extracted 1,860 items. Of these, 219 were excluded for reasons described above, and 548 were removed as duplicates, yielding 1,093 unique items. These items were consolidated into 243 indicators, organized in the framework seen in Figure 1. This framework contains 7 structural domains, and 5 process domains, further organized into sub-domains and categories.

Following the consolidation into 243 indicators, reduction was driven by two surveys of ASQ RG members. Responses to the first survey represented approximately 45% of the group. In this survey, we did not capture whether a response was submitted on behalf of several members of the resource group from the same organization. A second survey of the ASQ RG had a response rate of 50%, weighted to account for responses submitted on behalf of an organization. Table 1 lists the total number of indicators in each sub-domain during the two rounds of reduction. After the first survey, we removed indicators from categories receiving <25% of the vote, resulting in a 40% reduction in the number of indicators. Respondents in the second survey prioritized among the remaining indicators within selected categories, and a further 64 indicators (44%) were dropped. Notably, all indicators on composition and training of the health workforce were eliminated, as were indicators measuring the presence of administrative systems, facility outreach and equity, availability of other SRH services or referrals, and written informed consent. The ASQ RG responses also led to marked reduction in indicators to measure equipment (from 10 to 1), technical skills (from 32 to 12), and information provision (from 36 to 12).

**Table 1:** Stakeholder driven study indicator selection.

**Theme**	**Sub-theme**	**Total # of indicators**	**Retained in Round 1**	**Retained in Round 2**	**Added from qualitative findings**
1 - Infrastructure	1.1 Infrastructure	3	1	1	
	1.2 Facility cleanliness, comfort and privacy	4	2	2	1
	1.3 Administrative systems	7	0	0	
2 - Referral systems	2.0 Referral systems for abortion care	5	4	>4	
3 - Health Information Systems	3.1 Recordkeeping	24	4	3	
	3.2 Mechanisms for feedback	12	3	2	
4 - Supplies, Medicines and Equipment	4.1 Supplies	5	3	3	1
	4.2 Medicines	5	4	4	1
	4.3 Equipment	10	6	1	
	4.4 Supply chain and stockouts	7	5	4	
5 - Health Workers	5.1 Human resources	2	0	0	
	5.2 Supervision and management of clinicians/staff	7	0	0	
6 - Access	6.1 Outreach	2	0	0	
	6.2 Accessibility	9	9	6	1
	6.3 Equity	3	0	0	
7 - Continuum of care and service integration (structure)	7.1 Availability of contraceptive services	3	2	1	
	7.2 Availability of other SRH services or referrals	2	0	0	
8 - Technical competence	8.1 Pain Management	5	4	3	1
	8.2 Infection Prevention	12	12	5	1
	8.3 Technical Skills	32	32	12	4
9 - Decision making	9.1 Informed Consent	4	0	0	1
	9.2 Client choice (autonomy & absence of coercion)	5	3	3	4
	9.3 Support for decision	4	4	3	3
10 - Information provision	10.1 Information provision	36	24	12	3
	10.2 Bidirectional communication	4	2	1	4
	10.3 Client understanding	2	2	2	7
11 - Continuum of care & service integration (process)	11.1 Continuum of care & service integration	13	10	5	
12 - Client and Provider Interactions	12.1 Privacy and confidentiality	5	5	3	4
	12.2 Dignity & respect	8	4	3	3
	12.3 Trust & confidence	3	2	0	
**Total # of Indicators**		**243**	**147**	**83**	**39**

From the 83 remaining, indicators were collapsed or dropped if measuring similar constructs. Seventy-two indicators originating from the data collection tools of the 12 organizations remained.

**Figure 2:**
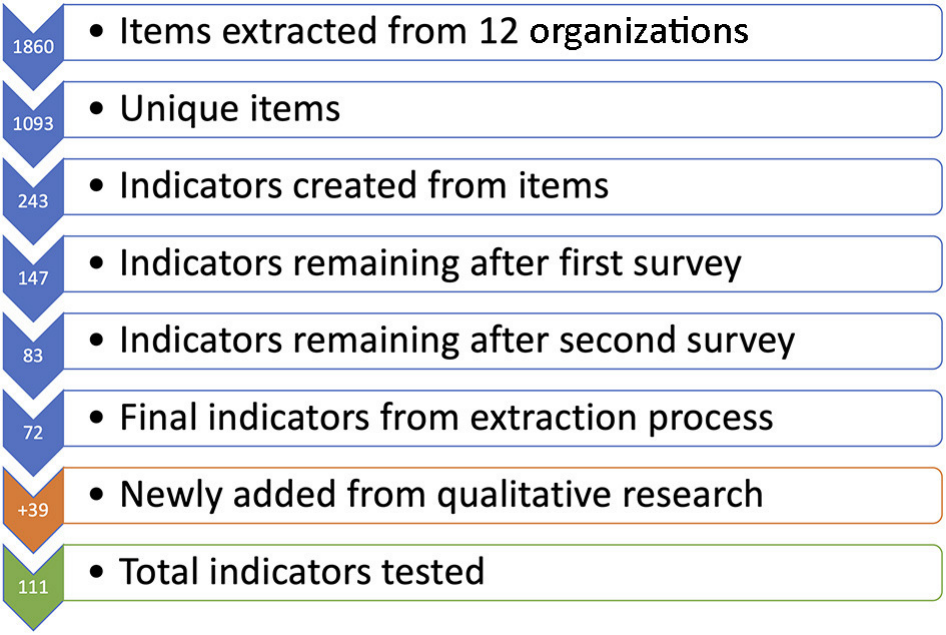
Indicator reduction process.

Analysis of the qualitative formative data collected in 2018 (25) led to the development and inclusion of 39 client-centered indicators. Some of these indicators were conceptually similar to existing indicators, but measured from the client's perspective. All were added to the remaining 72 indicators from the prior process. Figure 2 illustrates how the final list of 111 indicators was derived.

The ASQ RG was also engaged to identify the most important outcomes, against which indicators will be tested. Table 2 lists the 12 outcomes (9 client level; 3 facility level) selected as important to collect. Although numerous, the outcomes cover a variety of clinical and client experience outcomes considered crucial for assessing quality and require only 2 different data collection tools, and thus can be collected efficiently.

**Table 2:** Outcomes.

1	Client was treated with respect and kindness throughout the abortion process	Client
2	Client felt that they could cope with their pain	Client
3	Client felt they knew what to do if adverse events occurred	Client
4	Client was able to access follow-up or intervention for issues related to the abortion as desired	Client
5	Client knew their abortion was complete or had a plan for what to do	Client
6	Client was able to access ancillary services or referrals, such as contraceptive and STI/HIV services, if desired	Client
7	Client was no longer pregnant at 30 days	Client
8	Client experienced abortion-related infection	Client
9	Client would recommend the service to a friend	Client
10	Site has no deaths in the past year	Facility
11	Sites with expected range of SAEs in last year	Facility
12	Sites with clients turned away for abortion services	Facility

These indicators and outcomes were operationalized for data collection in three countries (Nigeria, Ethiopia and Bangladesh). Data were collected via client exit interview, provider interview, facility assessment and direct observation, as well as 30-day client follow-up survey to assess abortion outcomes.

As indicators were operationalized for data collection, the study team identified which indicators were applicable to which care settings. For example, an indicator on infrastructure for high-level disinfection or sterilization of instruments is applicable only to facilities offering surgical abortion, one about the site being clean and well-maintained is applicable to any physical site, and an indicator asking whether clients received or knew where to get necessary supplies to manage bleeding after abortion is applicable to all abortion clients. Table 3 lists the number of indicators in each domain and application for each care group. Overall, 47 of the 111 indicators tested (42%) were applicable to all abortion service settings, and an additional 15 indicators were applicable to services provided at a physical site. Seventy-five percent of the indicators were process indicators, and the majority of these revolve around the interactions between client and provider (domains of “decision making,” “information provision” and “client-provider interactions”). The ASQ Initiative intends to further reduce the indicators based on the three-country pilot test, and these results will be reported elsewhere.

**Table 3:** Applicable indicators by domain and care group.

**Domain**	**All abortion service settings**	**Facility only**	**Surgical only**	**Medical abortion only**	**Any physical site**	**Facility or hotline**
Total	47	31	9	6	15	3
1.Infrastructure	0	0	1	0	3	0
2.Referral Systems	1	0	0	0	0	0
3.Health Information Systems	2	1	0	0	0	0
4.Supplies, Medicine, Equipment	1	4	2	2	3	0
5.Health Workers	0	0	0	0	0	0
6.Access	7	0	0	0	0	0
7.Continuum of care (structural)	0	0	0	0	1	0
8.Technical competence	3	10	6	2	2	1
9.Decision Making	6	3	0	0	3	0
10.Information provision	18	7	0	2	2	2
11.Continuum of care (process)	1	2	0	0	0	0
12.Client-provider interactions	8	4	1	0	1	0

## Discussion and Conclusions

Universal indicators to assess healthcare performance have been established in many fields. While the realities of care may differ by location or circumstance, the moral obligation to provide quality care is universal. Our collaborative approach—involving key stakeholders at each stage of the process—demonstrates that in the provision of abortion services, there are myriad quality indicators already in use; that there is significant overlap among the different tools, with many key components of quality addressed in different tools using variations on the same language, and from different perspectives or with different data collection methods. We found that 56% of our shortlisted indicators (62 of 111) were nearly universally applicable to all abortion care settings. This implies that there are other types of indicators which are suited only to certain settings, and that quality assurance processes for abortion must be flexible in their approach. Indicators which are currently being collected across the 12 representative organizations span a wide range of topics, as evidenced by the theoretical framework we developed.

Nonetheless, we also identified key gaps in current abortion quality measurement. We were able to identify an additional 39 indicators from formative work centering the client experience, confirming a gap in available measures to achieve a holistic approach to measuring quality as guided by current thinking on what constitutes healthcare quality (24). Streamlining the number of indicators routinely used to assess abortion quality, while identifying those which are feasible to collect in low resource contexts, will allow for better monitoring of this already safe procedure (26). Even the 111 indicators resulting from this first part of the ASQ Initiative, and carried forward to our pilot study, would be burdensome, especially at low volume facilities (27).

Our approach may be limited in two ways. First, while our starting point yielded 1,093 unique data items, the organizations tools, and the contexts they represent, are not representative of all ways in which pregnant people seek abortions. For example, none of the providers were solely hotline based. Second, the participants of the ASQRG changed over time, although the represented organizations remained the same or expanded. This unavoidable lack of continuity given the length of the project may have impacted the understanding of participants on the project aims, or influenced their enthusiasm for participation. The number of participants in the ASQRG has been larger than found in many other Delphi or modified Delphi approaches.

We found the initial consensus building meeting, and initiation of the ASQRG to be an essential part of the project success. Although we did not predict the multiple ways in which the RG would be engaged during the project, establishing that all were working toward a shared goal from the start likely improved their engagement during the project.

Following from the work described in this paper, the analysis of the data from 2,000 abortion clients in Bangladesh, Ethiopia and Nigeria, representing first and second trimester care seeking at facilities, pharmacies and via hotline, will identify a reduced set of indicators associated with outcomes (28). The results of these associations will be further analyzed against the pre-determined set of criteria - actionable, valid, accurate, simple, timely and easy to collect - agreed to by the stakeholder group at the start of the ASQ Initiative.

The goal of the ASQ Initiative is to generate a concise minimum list of abortion service quality indicators which are broadly applicable, predictive of one or more outcomes of interest, and able to be collected in across a variety of care settings in low resource environments. We envision that the continued participation of the ASQ RG will facilitate dissemination and use of the metrics and tools. While some of our stakeholders have advanced quality measurement approaches, others are eager to use a pre-identified, minimum set of indicators to improve the quality of their services, and the health outcomes of people seeking abortions.

## Data Availability Statement

The raw data supporting the conclusions of this article will be made available by the authors, without undue reservation.

## Author Contributions

NC and EP organized the database. NC, EP, and SB performed the analysis. NC wrote the first draft of the manuscript. All authors contributed to conception, design of the study, interpretation of the analyses, manuscript revision, read, and approved the submitted version. All authors contributed to the article and approved the submitted version.

## Funding

Funding for this research has been provided by grants from the Children's Investment Fund Foundation and the David and Lucile Packard Foundation. Funders have collaborated in study oversight.

## Conflict of Interest

The authors declare that the research was conducted in the absence of any commercial or financial relationships that could be construed as a potential conflict of interest.

## Publisher's Note

All claims expressed in this article are solely those of the authors and do not necessarily represent those of their affiliated organizations, or those of the publisher, the editors and the reviewers. Any product that may be evaluated in this article, or claim that may be made by its manufacturer, is not guaranteed or endorsed by the publisher.

## References

[1] National National Academies of Sciences Engineering and Medicine. The Safety and Quality of Abortion Care in the United States. National Academies Press. (2018). p. 223. 29897702

[2] Donabedian A. The Quality of Care. JAMA. (1988) 260:1743–8. 10.1001/jama.260.12.1743 3045356

[3] Institute of Medicine. Crossing the Quality Chasm: A New Health System for the 21st Century. Washington, DC: The National Academies Press (2001). 25057539

[4] World Health Organization. Quality of Care: A Process for Making Strategic Choices in Health Systems. Geneva: World Health Organization (2006). Available online at: https://apps.who.int/iris/handle/10665/43470

[5] Benson J. Evaluating abortion-care programs: old challenges, new directions. Stud Fam Plann. (2005) 36:189–202. 10.1111/j.1728-4465.2005.00061.x 16209177

[6] Darney BG Powell B Andersen K Baum SE Blanchard K Gerdts C . Quality of care and abortion: beyond safety. BMJ Sex Reprod Health. (2018) 44:159–60. 10.1136/bmjsrh-2018-200060 29972364 PMC6225511

[7] Darney BG Kapp N Andersen K Baum SE Blanchard K Gerdts C . Definitions, measurement and indicator selection for quality of care in abortion. Contraception. (2019) 100:354–9. 10.1016/j.contraception.2019.07.006 31356772

[8] Filippi V Dennis M Calvert C Tunçalp Ö Ganatra B Kim CR . Abortion metrics: a scoping review of abortion measures and indicators. BMJ Global Health. (2021) 6:e003813. 10.1136/bmjgh-2020-003813 33514592 PMC7849886

[9] Shekhar C Sundaram A Alagarajan M Pradhan MR Sahoo H. Providing quality abortion care: findings from a study of six states in India. Sex Reprod Healthc. (2020) 24:100497. 10.1016/j.srhc.2020.100497 32036281

[10] Owolabi O Riley T Otupiri E Polis CB Larsen-Reindorf R. The infrastructural capacity of Ghanaian health facilities to provide safe abortion and post-abortion care: a cross-sectional study. BMC Health Serv Res. (2021) 21:1104. 10.1186/s12913-021-07141-5 34654428 PMC8520210

[11] Otsea K Benson J Alemayehu T Pearson E Healy J. Testing the safe abortion care model in Ethiopia to monitor service availability, use, and quality. Int J Gynaecol Obstet. (2011) 115:316–21. 10.1016/j.ijgo.2011.09.003 22019316

[12] Dennis A Blanchard K Bessenaar T. Identifying indicators for quality abortion care: a systematic literature review. J Fam Plan Reprod Healthcare. (2017) 43:7–15. 10.1136/jfprhc-2015-101427 27172880

[13] Tuncalp O Were WM MacLennan C Oladapo OT Gulmezoglu AM Bahl R . Quality of care for pregnant women and newborns-the WHO vision. BJOG. (2015) 122:1045–9. 10.1111/1471-0528.13451 25929823 PMC5029576

[14] Larson E Sharma J Bohren MA Tunçalp Ö. When the patient is the expert: measuring patient experience and satisfaction with care. Bull World Health Organ. (2019) 97:563–9. 10.2471/BLT.18.225201 31384074 PMC6653815

[15] McLemore MR Desai S Freedman L James EA Taylor D. Women know best—findings from a thematic analysis of 5,214 surveys of abortion care experience. Womens Health Issues. (2014) 24:594–9. 10.1016/j.whi.2014.07.001 25442704

[16] Sudhinaraset M Landrian A Afulani PA Phillips B Diamond-Smith N Cotter S. Development and validation of a person-centered abortion scale: the experiences of care in private facilities in Kenya. BMC Womens Health. (2020) 20:208. 10.1186/s12905-020-01071-w 32950052 PMC7501655

[17] Altshuler AL Whaley NS. The patient perspective: perceptions of the quality of the abortion experience. Curr Opin Obstet Gynecol. (2018) 30:407–13. 10.1097/GCO.0000000000000492 30299320

[18] Cotter SY Sudhinaraset M Phillips B Seefeld CA Mugwanga Z Golub G . Person-centred care for abortion services in private facilities to improve women's experiences in Kenya. Cult Health Sex. (2021) 23:224–39. 10.1080/13691058.2019.1701083 32105189

[19] Boulkedid R Abdoul H Loustau M Sibony O Alberti C. Using and reporting the delphi method for selecting healthcare quality indicators: a systematic review. Wright JM, editor PLoS ONE. (2011) 6:e20476. 10.1371/journal.pone.0020476 21694759 PMC3111406

[20] Fink A Kosecoff J Chassin M Brook RH. Consensus methods: characteristics and guidelines for use. Am J Public Health. (1984) 74:979–83. 10.2105/AJPH.74.9.979 6380323 PMC1651783

[21] Campbell SM Braspenning J Hutchinson A Marshall M. Research methods used in developing and applying quality indicators in primary care. Qual Saf Health Care. (2002) 11:358. 10.1136/qhc.11.4.358 12468698 PMC1758017

[22] Voerman GE Calsbeek H Maassen ITHM Wiegers TA Braspenning J. A systematic approach towards the development of a set of quality indicators for public reporting in community-based maternity care. Midwifery. (2013) 29:316–24. 10.1016/j.midw.2012.01.012 23357096

[23] Rubin HR Pronovost P Diette GB. Methodology matters. From a process of care to a measure: the development and testing of a quality indicator. Int J Qual Healthcare. (2001) 13:489–96. 10.1093/intqhc/13.6.489 11769752

[24] Akachi Y Kruk ME. Quality of care: measuring a neglected driver of improved health. Bull World Health Organ. (2017) 95:465–72. 10.2471/BLT.16.180190 28603313 PMC5463815

[25] Jacobson LE Ramirez AM Bercu C Katz A Gerdts C Baum SE. Understanding the abortion experiences of young people to inform quality care in Argentina, Bangladesh, Ethiopia, and Nigeria. Youth Soc. (2021). 10.1177/0044118X211011015

[26] World Health Organization. Safe Abortion: Technical and Policy Guidance for Health Systems. 2nd ed. Geneva: World Health Organization (2012). 23700650

[27] Leisher S Sprockett A Longfield K Montagu D. Quality Measurement in Family Planning: Past, Present, Future: Papers from the Bellagio Meeting on Family Planning Quality. Oakland, CA: Metrics for Management. (2015).

[28] Metrics for Management Ipas Ibis Reproductive Health. Measuring Abortion Service Quality. Available online at: https://asq-initiative.org/wp-content/uploads/2021/07/asq-measuring_web.pdf (accessed May 17, 2022).

